# Tumour Mutational Burden and Its Relationship with Clinical Outcomes in Locally Advanced and Recurrent/Metastatic Adenoid Cystic Carcinoma with and Without NOTCH Pathway Activation

**DOI:** 10.3390/cancers18121930

**Published:** 2026-06-13

**Authors:** Karan Patel, Joseph Edward Haigh, Samuel Rack, Hitesh Mistry, Emily Heathcote, Guy N. J. Betts, Kevin Harrington, Robert Metcalf

**Affiliations:** 1 The Christie NHS Foundation Trust, Wilmslow Road, Manchester M20 4BX, UKjoseph.haigh3@nhs.net (J.E.H.);; 2 School of Health Sciences, The University of Manchester, Oxford Road, Manchester M13 9PL, UK; 3 Manchester University NHS Foundation Trust, Manchester M13 9WL, UK; 4 The Institute of Cancer Research, National Institute of Health Research Biomedical Research Centre, London SW7 3RP, UK; 5 The Royal Marsden NHS Foundation Trust, Downs Road, Sutton, Surrey SM2 5PT, UK

**Keywords:** adenoid cystic carcinoma, tumour mutation burden, NOTCH

## Abstract

Background/Objectives: Adenoid cystic carcinoma (ACC) is a heterogenous disease and defining aggressive subtypes to inform surveillance and treatment strategies remains clinically pertinent. NOTCH pathway-activated ACC is associated with worse outcomes. We describe the distribution of tumour mutational burden (TMB) and characterise its relationship with NOTCH gain-of-function (GoF) mutation and survival in locally advanced or recurrent/metastatic (LA-R/M) ACC. Methods: 124 tumours from the UK NHS (NHS group) and 139 tumours from cBioPortal; MSK metTropism (MSK group) were evaluated as independent cohorts. TMB (mut/Mb) was calculated either by F1CDx or its precursor FoundationOne NGS in the NHS group, or MSK-IMPACT NGS in the MSK group. NOTCH1/2 mutations were classed as GoF if predicted to disrupt the NRR/PEST domains. Overall survival (OS) was measured from diagnosis of unresectable LA-R/M ACC. Results: Median TMB was 1.26 mut/Mb (IQR 0–2.52) in the NHS group and 1.96 (IQR 0.87–3.46) in the MSK group. NOTCH1/2 GoF mutation was seen in 16/124 (13%) in the NHS group and 22/139 (16%) in the MSK group. Median TMB for tumours with NOTCH1/2 GoF mutation was 2.52 mut/Mb (IQR 1.82–3.84) in the NHS group and 4.38 mut/Mb (IQR 3.33–4.89) in the MSK group. For tumours with NOTCH1/2 GoF mutation, median OS reduced with TMB > median of 2.52 mut/Mb (0.7 yrs vs. 2.6 yrs, *p* = 0.02) in the NHS group. Conclusions: LA-R/M ACC has a low TMB profile overall. Median TMB was higher in NOTCH-activated ACC in both the NHS and MSK groups and TMB may have value in further stratifying patients with LA-R/M ACC.

## Simple Summary

Adenoid cystic carcinoma (ACC) is a heterogeneous disease that may benefit from better stratification of aggressive subtypes. We analysed two patient groups with incurable ACC to describe the number of different mutations per sample (tumour mutational burden) and its relationships with genetic subgroups and survival. We found that tumour mutational burden is generally low in ACC and typically higher for tumours with NOTCH gain-of-function mutations. Tumour mutational burden may be useful in further stratifying patients with ACC.

## 1. Introduction

Adenoid cystic carcinoma (ACC) is a rare cancer [1] arising from the ductal epithelium and myoepithelium of salivary and extra-salivary secretory glands [2,3]. The disease is commonly slow growing, but recurrence rates are high [4,5], and a proportion of patients have a more rapidly progressive disease course [6,7]. Response rates to chemotherapies [8] and immunotherapies [9,10,11] are low, and there is no standard of care in the non-curative setting [12,13]. Consequently, key studies have scrutinised the ACC mutanome to determine genetic drivers that help explain the biological basis of aggressive subtypes and identify druggable targets. Oncogenic mutations previously elucidated include MYB gene rearrangement, NOTCH gain-of-function (GoF) mutations, TP53 loss-of-function (LoF) alterations and LoF mutations in genes involved in chromatin remodelling [14,15,16,17,18,19]. Additionally, these gene mutations are associated with poorer outcomes in ACC [20,21], with alterations triggering the NOTCH-signalling pathway yielding the worst prognosis [21].

Tumour mutational burden (TMB), denoted as the number of mutations per megabase within a tumour, is a complex biomarker potentially reflective of oncogenic functioning and tumour immunogenicity [22,23]. There are multiple ways of defining TMB, and TMB values substantially differ across cancer types in pan-cancer analyses [22,24,25]. Although studies have depicted a complicated relationship between TMB and prognosis [26,27,28], higher TMB is often predictive of longer survival with immunotherapy [29,30]. Notably, the response rates of tumours with a TMB ≥ 10 mutations/megabase (mut/Mb) (TMB-High) to pembrolizumab immunotherapy in a Keynote-158 sub-study [31] led to FDA approval of pembrolizumab for such tumours that have progressed after one line of therapy and have no other satisfactory alternative options [32]. Median TMB is reported to be low in ACC (0.34–1.7 mut/Mb) [15,33,34,35], but comprehensive distribution data are lacking, and the association between TMB and outcomes remains undetermined.

Thus, we sought to describe the distribution of TMB values in two independent patient cohorts with unresectable locally advanced (LA) or recurrent/metastatic (R/M) ACC, and to determine the relationship between TMB, key genetic alterations and clinical outcomes.

## 2. Materials and Methods

### 2.1. Study Population and Ethical Approvals

Two patient cohorts with LA-R/M ACC were independently evaluated. For the first cohort, patients were prospectively recruited between April 2017 and January 2022 through a single UK cancer centre (NHS cohort). Subjects gave written informed consent for an ethically approved study authorising the collection and analysis of their clinical and genetic information. The study was granted research ethics approval under the MCRC Biobank Research Tissue Bank Ethics (NHS NW Research Ethics Committee 18/NW/0092) and was performed in accordance with the Declaration of Helsinki. Clinical data were retrospectively reviewed, including dates of diagnosis, recurrence, and last follow-up, to calculate the overall survival. For the second cohort, matched clinical-genomic data for 139 patients with LA-R/M ACC were obtained from the online open-source database cBioportal MSK MetTropism (MSK cohort). Patients gave written informed consent to a clinical study allowing analyses of clinical and genomic data (NCT01775072), which was approved by the Memorial Sloan Kettering Cancer Centre Institutional Review Board [25]. Clinical data, as outlined for the NHS cohort, were acquired from cBioPortal and retrospectively reviewed for the MSK cohort.

### 2.2. DNA-Based Next-Generation Sequencing (NGS), TMB Estimation and Microsatellite Status

For the NHS cohort, DNA extracted from fixed-formalin paraffin-embedded (FFPE) tumour underwent NGS using either the FoundationOne^®^ CDX assay (F1CDx) (Foundation Medicine, Cambridge, MA, USA) or its predecessor FoundationOne^®^ (F1 LDT). For the F1Cdx assay, tumour DNA underwent hybrid capture and sequencing using the HiSeq 4000 platform (Illumina^®^, San Diego, CA, USA) to high uniform depth (targeting > 500× median coverage with >99% of exons at coverage > 100×) in order to detect insertion and deletion alterations (indels), and copy number alterations (CNAs) in 324 genes. TMB estimates were obtained by counting all synonymous and non-synonymous variants present at 5% allele frequency or higher, and filtering out potential germline variants (either via published databases or via in silico data) and driver mutations. The mutation number was divided by the coding region corresponding to the number of total variants counted, or 793 kilobases (kb), to obtain estimates in mut/Mb [36]. With the F1 LDT assay, tumour DNA was sequenced using the HiSeq 2000 platform (Illumina^®^, San Diego, CA, USA to high uniform depth (targeting > 500× median coverage with >99% of exons at coverage > 100×) in order to detect indels and CNAs in 315 genes [37]. TMB estimates from F1CDx and F1 LDT have shown excellent concordance using linear regression analyses [38].

In the MSK cohort, all tumours underwent NGS with three iterations of the Memorial Sloan Kettering Integrated Molecular Profiling of Actionable Cancer Targets (MSK-IMPACT) clinical sequencing assay, using 341-, 410-, and 468-gene panels, respectively. Matched normal DNA was acquired from blood samples [25,39]. TMB estimates (mut/Mb) from MSK-IMPACT were calculated by dividing the number of non-synonymous and oncogenic driver mutations by the total genomic area evaluated, varying according to the assay version used. Germline alterations were excluded by subtracting matched normal samples [23].

### 2.3. Classification of NOTCH GoF Mutation, TP53 LoF and Chromatin Remodelling Gene LoF

Genomic panels in all patients in both cohorts were reviewed to identify alterations in NOTCH1/2/3, TP53, and key genes reported to be involved in chromatin regulation (CREBBP/EP300, KDM6A, SETD2, KMT2C, KMT2D, ARID1A) that had a predicted prevalence of >1% in ACC. ARID1B LoF mutations and KMT2C LoF were not included in analyses as no functional ARID1B or KMT2C mutations were found in the NHS cohort. NOTCH mutations were classed as gain-of-function if predicted to disrupt the heterodimerization-negative regulatory region (NRR) or the proline-glutamic acid-serine-threonine-rich (PEST) domain as previously described [21]. Genetic alterations in TP53 and chromatin remodelling genes were classed as loss-of-function based on assessment using the Genome Aggregation Database (gnomAD) and Catalogue of Somatic Mutations in Cancer (COSMIC) databases. Gene alterations were defined as pathogenic if they were ascribed a FATHMM score over 0.9 in COSMIC, described as “likely pathogenic” in ClinVar/GnomAD, or stated to be “likely oncogenic” in OncoKB™ [20].

### 2.4. Statistical Analyses

The Mann–Whitney U test was used to compare the distributions and median values of the clinical characteristics between the two cohorts. The Wilcoxon signed-rank test was used for paired samples within the genetic subgroup analysis. The other sub-analyses were compared using the Mann–Whitney U test for two groups or the Kruskal–Wallis test for comparisons with more than two groups. Overall survival (OS) was calculated using Kaplan–Meier analysis and log-rank tests. OS was defined as the time from diagnosis of unresectable LA or R/M ACC to death of any cause. Patients who were alive at the data cut-off were censored at the date of the last follow-up. Log-rank *p*-values were two-sided; *p* ≤ 0.05 was accepted as statistically significant. Hazard ratios were calculated using non-linear Cox proportional hazard models and plotted using a spline function. Spearman correlation coefficients were calculated to observe for correlation between TMB and OS. Statistical analyses were conducted in R (v4.1.3 and v4.4.3).

## 3. Results

### 3.1. Patient and Tumour Characteristics

In the NHS cohort, 124 tumour samples that underwent targeted DNA-based NGS via Foundation Medicine assays had TMB data; 139 tumour specimens with relevant clinic-genomic data were evaluated in the MSK cohort. Clinical characteristics of both cohorts are summarised in Table 1. Median age at diagnosis of unresectable, LA-R/M disease was 58.2 years (range: 18.5–86 years) in the NHS cohort and 53.7 years (range: 22.8–90 years) in the MSK cohort. Tumour samples were from patients with ACC arising from the major salivary glands, where this information was available, in 41% (NHS) and 30% (MSK) of cases. In cases where the biopsy location was known, 47/95 (50%) tumour samples were taken from metastatic sites in the NHS group, whereas 100/139 (72%) specimens in the MSK group were procured from metastases, a significantly higher proportion. There was a higher proportion of tumour samples from patients with bone and CNS metastases in the MSK cohort compared with the NHS cohort, and the NHS cohort had significantly more patients with metastatic disease in the lungs only, but the overall distribution of metastatic disease was not significantly different. No tumour specimens in either cohort demonstrated microsatellite instability (MSI).

### 3.2. TMB Distribution and Overall Survival in Whole Cohort

We first evaluated the TMB distributions between each cohort to evaluate for any differences, given the variation in demographics and panel assays used. Median TMB in the NHS cohort was 1.26 mut/Mb (IQR 0–2.52 mut/Mb) and 1.96 mut/Mb (IQR 0.87–3.46 mut/Mb) in the MSK cohort. Only one tumour (0.8%) in the NHS cohort and no tumours in the MSK cohort had a TMB of ≥10 mut/Mb. The TMB-High tumour specimen in the NHS cohort was taken from a regional recurrence arising from a high-grade submandibular primary and harboured a BRCA1 Associated Protein 1 gene mutation, a non-activating NOTCH2 mutation, and a TERT promoter gene alteration, but not any functional mutations in NOTCH, TP53 or any of the chromatin remodelling genes evaluated. TERT mutations have previously been described to be mutually exclusive with NOTCH1 gene alterations as well as MYB/MYBL1 fusions [15].

**Figure 1:**
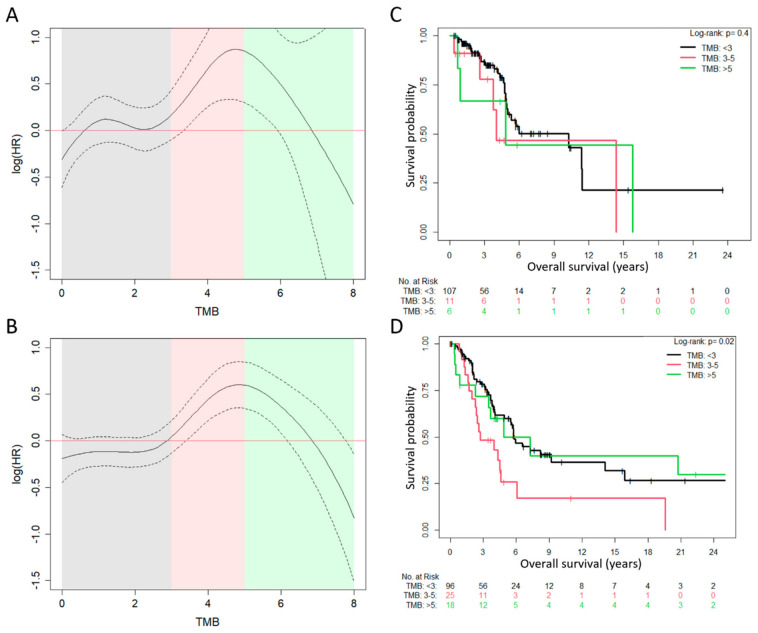
Non-linear Cox proportional hazards model showing the log of the hazard ratio (log(HR)) by tumour mutation burden (TMB) for the NHS cohort (**A**) and MSK cohort (**B**). The log(HR) is displayed with a solid line. Confidence intervals are displayed with dashed lines. TMB was categorised into <3 (grey), 3–5 (red) and >5 (green). Kaplan–Meier plots showing overall survival by TMB category are shown for the NHS cohort (**C**) and MSK cohort (**D**). Censored subjects are shown with a vertical line.

Analysis of TMB as a continuous variable showed that TMB had a non-linear relationship with survival in the NHS and MSK cohorts (Figure 1A,B). TMB values of 3–5 mut/Mb were associated with an increased risk of death compared with TMB values higher and lower than this in both cohorts. However, wide confidence intervals were observed in the NHS cohort. TMB was then examined as a tiered variable, using the slope of the log-adjusted hazard ratio curves to inform categories. This demonstrated a significantly reduced median OS in tumours with TMB values of 3–5 mut/Mb compared to those with TMB values of <3 mut/Mb and ≥5 mut/Mb in the MSK cohort (median OS of 4.0 years for tumours with TMB 3–5 mut/Mb, compared to 6.0 years and 4.9 years for tumours with TMB values < 3 mut/Mb and ≥5 mut/Mb, respectively (*p* = 0.003) (Figure 1C,D). However, this effect was not observed after excluding tumours with NOTCH1/2 GoF mutations.

### 3.3. TMB Distribution and Overall Survival in NOTCH-Activated Subgroup

As NOTCH GoF mutations are significantly associated with high-grade disease and poorer survival [21,40], we then examined the relationship between TMB and NOTCH-activated ACC. 16 (13%) tumours in the NHS cohort and 22 (16%) tumour specimens in the MSK cohort had activating mutations affecting NOTCH1/2. There were no significant differences in the rate of NOTCH GoF mutations between metastatic or primary biopsy sites in either the NHS cohort (*p* = 0.16) or MSK cohort (*p* = 0.81). In the NHS cohort, median TMB was 2.52 for the tumour samples with NOTCH1/2 GoF mutations. Median TMB was significantly higher in the tumours with NOTCH1/2 GoF compared to tumours without NOTCH1/2 GoF mutations (2.52 mt/mb vs. 1.26 mt/mb, *p* = 0.003). In the MSK cohort, median TMB was 4.38 mut/Mb for tumours with NOTCH1/2 GoF mutation (IQR 3.33–4.89).

**Figure 2:**
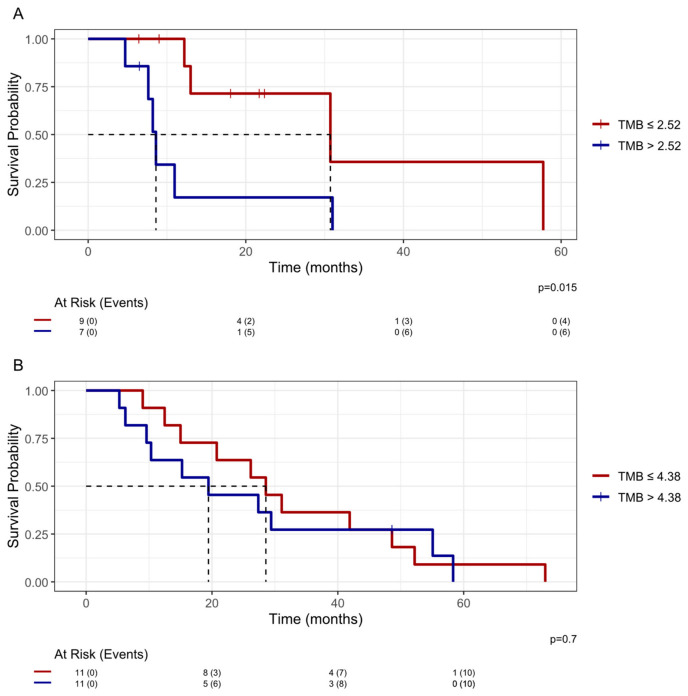
Kaplan–Meier analyses showing overall survival in months for NOTCH-activated ACC in the NHS cohort (**A**) and MSK cohort (**B**). Censored individuals are displayed with a vertical line. The median is shown with dashed lines.

For tumour samples with NOTCH1/2 GoF mutation in the NHS group, median OS decreased with higher TMB. Median OS was 0.7 years in NOTCH1/2-mutated tumour with a TMB higher than the median TMB of 2.52 mut/Mb compared with 2.6 years in NOTCH1/2 activated-tumour samples with TMB values lower than the median (*p* = 0.02) (Figure 2A). In the MSK cohort, the same trend towards shorter OS was seen in tumours with NOTCH1/2-activting mutations, but this did not reach statistical significance (median OS 1.6 years vs. 2.4 years, *p* = 0.7) (Figure 2B).

### 3.4. TMB Distribution and Overall Survival in Other Mutational Subgroups

For both cohorts, the tumour mutation subgroup with the highest median TMB was SETD2 LoF alterations (2.63 mut/Mb [IQR 0.95–4.00 mut/Mb] in the NHS cohort and 6.92 mut/Mb [IQR 6.23–6.92 mut/Mb] in the MSK cohort). However, the incidence of SETD2 mutations was low at just 3% and 2%, respectively (Table 2). Median TMB was 2.52 for the tumour samples with TP53 LoF mutations, CREBBP/EP300 LoF mutations, KDM6A LoF mutations and ARID1A mutations in the NHS cohort. Similar to tumours with NOTCH1/2 GoF mutations, median TMB was significantly higher in ACC with TP53 LoF mutations compared to TP53 wild-type disease at 2.52 mt/MB vs. 1.26 mt/MB in the NHS (*p* = 0.01) and 3.33 mt/MB vs. 1.84 mt/MB in the MSK cohorts (*p* = 0.002). In the MSK cohort, the second-highest median TMB was observed in tumours expressing functional mutations in CREBBP and its paralog EP300 (5.53 mut/Mb [IQR 3.3–6.05]) (Table 2). All tumour mutation subgroups in the MSK cohort, except the KMT2D LoF mutation, had significantly higher TMB values when compared independently with tumours without the respective LoF mutation (*p*-values < 0.05). There were no significant differences between the median TMB values of the 7 tumour mutation subgroups in either cohort.

There was no significant association between TMB and OS in any of the mutational subgroups analysed, except for NOTCH1/2 ACC tumour subgroups in either cohort. Survival analyses were not performed for tumours with SETD2 LoF mutations because of the low numbers present in both cohorts.

### 3.5. TMB Distribution and Overall Survival According to Biopsy Site and Metastatic Burden

**Figure 3:**
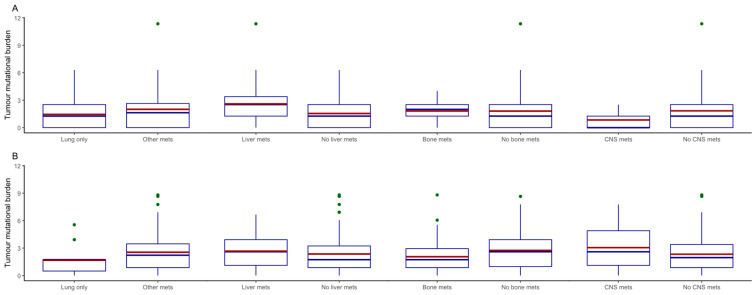
TMB distribution by metastatic site for the NHS cohort (**A**) and MSK cohort (**B**). The median is displayed as a bold blue line. The mean is displayed as a bold red line. Outliers are displayed in green.

Previous research has demonstrated that TMB may vary between primary and metastatic sites in some cancers [41,42]. As a result, we investigated the associations between TMB and biopsy site and no significant differences were seen in either the NHS cohort (*p* = 0.1) or the MSK cohort (*p* = 0.6). We then examined for differences in TMB according to disease burden, and no statistically significant differences were seen in TMB values between tumours from patients with lung-only metastatic disease compared with tumour specimens biopsied from patients with the presence of non-lung distant metastatic disease in either cohort. However, in the NHS cohort, median TMB was significantly higher in tumours from patients with liver metastases than patients with no liver involvement (2.52 mut/Mb vs. 1.26 mut/mb, *p* = 0.01 (Figure 3A). In the MSK cohort, median TMB was non-significantly higher in tumour specimens from patients with liver metastases than those without (2.59 mut/Mb vs. 1.73 mut/Mb, *p* = 0.12). Median TMB was significantly higher where bony metastases were present (2.59 mut/Mb vs. 1.73 mut/mb, *p* = 0.02) (Figure 3B). No significant correlation was observed between median TMB values and the number of organs affected by metastatic disease. Sub-analyses showed little correlation between TMB and OS in ACC tumour subgroups with bone or liver metastases in either the NHS or MSK cohort. This was the case in all tumour specimens regardless of survival status.

## 4. Discussion

In our study, the median TMB was low in both cohorts using NGS (1.26 mut/mb in the NHS cohort and 1.96 mut/mb in the MSK cohort). One tumour sample (0.8%) in the NHS cohort and none in the MSK cohort had a TMB ≥ 10 mut/Mb, the threshold for classification as TMB-high for tumour-agnostic FDA approval for the use of pembrolizumab. Overall, this is consistent with the existing data. Ho et al. [15] and Dou et al. [34] reported median values of TMB of 0.34 mut/Mb and 0.85 mut/Mb using WES in their respective studies of ACC. Dou et al. also noted that 3 (4%) of the 75 head and neck ACC samples analysed had TMB values ≥ 10 mut/Mb; one of which had a TMB of 230.33 mut/Mb. A study examining salivary, tracheal and breast ACC using a Foundation Medicine targeted panel found that the median TMB was 1.7 mut/Mb for all three sites. In this study, 0.3% of salivary gland ACC tumours and 6% of breast ACC tumour samples had a TMB of ≥10 mut/Mb [35]. Another study observed that one out of 154 (0.7%) ACC tumours had a TMB ≥ 10 mut/Mb in their study using a Foundation Medicine NGS assay [17].

Although TMB did not significantly correlate with survival in the whole NHS cohort, patients with tumours possessing an intermediate TMB value of 3–5 mut/Mb had the worst prognosis in the MSK cohort. Riviere et al. recorded a parabolic relationship with TMB and overall survival in a pan-tumour study of immunotherapy-naïve patients [27]. The reduced survival in the MSK subcohort possessing tumours with TMB of 3–5 mut/Mb in our study may reflect the high proportion of these same tumours also harbouring NOTCH1/2 GoF mutations (16 of 25 tumours; 64%). The NOTCH mutations may be driving the shorter survival rather than the TMB in this subcohort, which is supported by the subcohort no longer showing a significantly shorter survival once removing patients with NOTCH GoF mutations.

ACC with NOTCH GoF mutations in the PEST or NRR domains represent a distinct subtype of ACC associated with attenuated progression-free and overall survival times [21,40,43,44]. Feeney et al. reported that the median OS from time of R/M disease was 1.9 years for NOTCH-activated ACC compared with 9.6 years for tumours without NOTCH activation (*p* < 0.0001). In this study, Median TMB was significantly higher in tumours with NOTCH1/2 GOF than in tumours without. In addition, higher TMB values were associated with worse survival in ACC exhibiting NOTCH1/2 GoF mutations in both the NHS and MSK cohorts, and this was statistically significant for the NHS cohort. Here, median OS was 0.7 years in the NHS cohort and 1.6 years in the MSK cohort for NOTCH-activated tumours with TMB values greater than the median. Notably, tumours with NOTCH GoF mutation commonly have functional mutations affecting TP53 and chromatin remodelling genes. Mutations in TP53 and the chromatin remodelling genes KDM6A and SETD2 are all associated with poorer outcomes [20,21]. In this study, 12 of 16 (75%) tumours in the NHS cohort and 14 of 22 (64%) in the MSK cohort harboured NOTCH1/2 GoF as well as functional mutations affecting TP53 and/or the chromatin remodelling genes. Data observing TMB and mutation type in ACC are limited, but Ferrarotto et al. observed that median TMB was higher in ACC-I tumour subtype than ACC-II disease using deep-targeted exome sequencing (2.3 mut/Mb vs. 0.7 mut/Mb, *p* = 0.004); ACC-I had significantly more mutations in NOTCH1, CREBBP and EP300 [43]. Furthermore, Ross et al. stated that salivary gland pathologies with <20% rates of TP53 mutations had significantly lower TMB than histopathologies with TP53 mutation rates of >40% [17].

There were no statistically significant differences between TMB values of tumours sequenced from primary or metastatic sites in either cohort. A pan-cancer study published in 2019, which included salivary gland tumours, found that TMB was significantly higher in metastatic sites than in primary tumours on grouped analysis [41]. However, other studies have observed that the hierarchy of TMB values between primary tumours and metastases differed according to cancer type [25,42].

Whole exome sequencing (WES) provides a standardised method of measuring TMB. However, targeted panels are often used in research and clinical settings because of their reduced cost and turnaround time [23]. Differences in median TMB values described in ACC between studies using WES [15,39] compared to our findings may be explained by the use of WES from fresh tumour samples to calculate TMB rather than NGS on FFPE tissue. Additionally, different panel sizes, gene content, and filtering methods for germline alterations between the Foundation Medicine NGS and MSK-impact assays are likely to contribute to the variations in TMB seen between the NHS and MSK cohorts [45].

Previous research has characterised ACC as poorly immunogenic. Relevant translational studies have shown that ACC tumour tissue often displays low levels of tumour-infiltrating lymphocytes (TILs), natural killer cells, T-cell signature RNA gene expression, and immune checkpoint molecules such as CTLA4, LAG3, TIM, and TIGIT [33,46,47,48]. Although ACC commonly exhibits PD-L2 on IHC, the incidence of PD-L1 expression is low [49]. Furthermore, high numbers of immunosuppressive cells are observed in ACC tumour, including myeloid-derived suppressor cells, and M2 tumour-associated macrophages [33,50,51]. These findings provide evidence of the possible causes for the lack of efficacy seen with CTLA4 and PD-1/PD-L1 inhibition [9,10,11,52]; sub-analyses of Keynote-158 found that only 2 of 59 patients with ACC had a partial response to pembrolizumab [11].

The main limitations of this study are the use of retrospective data and lower participant numbers, particularly with specific gene mutations, which is due to the rare nature of the disease type. In addition, although clinical data ascertaining patient demographics and disease spread are relatively comprehensive, due to the retrospective nature of this dataset, treatment details are incomplete, and therefore, we were not able to analyse any impact of treatment on survival outcomes. As such, further prospective studies in larger well-controlled cohorts remain a top priority for future research.

ACC is known to be predominantly driven by MYB or MYBL1 fusions, most commonly MYB::NFIB fusions. Recent data suggest that the type of MYB fusion detected by fluorescent in situ hybridisation (FISH) may influence survival outcomes in ACC [53]. However, MYB fusion FISH is not currently standard of care for ACC patients, and insufficient data were available to include in the analysis of this study.

There has been limited investigation into targeting specific gene alterations in ACC. NOTCH GoF has been evaluated as a candidate drug target in the ACCURACY study, although there was no clear signal of efficacy [54]. MYB fusions are also of therapeutic relevance, with ongoing studies of MYB-degrader therapies. These have recently posted results suggesting a signal of efficacy [55,56]. No other genetic targets have been formally tested.

## 5. Conclusions

This study described TMB values in a large cohort of 263 ACC patients and shows that NOTCH-activated ACC, which already possesses a poorer prognosis, may be further stratified by TMB. This may be helpful in directing clinical management, including timing of interval surveillance imaging and clinician-patient discussions regarding earlier initiation of systemic therapies, and warrants validation in a prospective study. A helpful next step would be to investigate total, clonal and persistent TMB in a larger number of tumours with NOTCH GoF mutation, as well as mutations affecting the broader NOTCH signalling pathway.

## Figures and Tables

**Table 1:** Baseline patient and tumour characteristics. Characteristics that differ significantly between groups are marked with *.

Characteristic		NHS Cohort, *n* = 124 (%)	MSK Cohort, *n* = 139 (%)	*p*-Value (Sig. < 0.05)
Age at recurrence (years, range)		58.2 (18.5–86.0)	53.7 (22.8–90)	0.06
Sex	Female	69 (55.6)	69 (49.6)	0.40
	Male	55 (44.4)	70 (50.4)	
Salivary gland	Major	51 (41.1.)	42 (30.2)	0.99
	Minor	72 (58.1)	57 (41)	
	Unknown	1 (0.8)	40 (28.8)	
Biopsy site	Primary	48 (37.5)	39 (28.1)	<0.001 *
	Metastasis	47 (38.3)	100 (79.1)	
	Unknown	29 (24.2)	0 (0)	
Microsatellite instability	Stable	95	119	0.59
	Indeterminate	0	2	
	Unknown	29	18	
Metastatic pattern	Lung only	43 (34.7)	11 (7.9)	<0.001 *
	Non-lung only	64 (51.6)	128 (92.1)	
Site of metastasis	Lung	95 (76.6)	112 (80.6)	0.08
	Liver	31 (25)	53 (38.1)	
	Bone	25 (20.2)	84 (60.4)	
	CNS	3 (2.4)	28 (20.1)	

**Table 2:** Median TMB and distribution for key mutations in NHS and MSK cohorts.

Mutations	NHS Cohort, *n*-124		MSK Cohort, *n*-139		*p*-Value (Sig. < 0.05)
	No. (%)	Median (IQR), mut/Mb	No. (%)	Median (IQR), mut/Mb	
*NOTCH 1/2* GoF mutation	16 (13)	2.52 (1.82–3.84)	22 (16)	4.38 (3.33–4.89)	0.60
*TP53* LoF mutation	15 (12)	2.52 (1.89–3.78)	17 (12)	3.33 (2.59–4.89)	1
*KDM6A* LoF mutation	12 (10)	2.52 (1.26–2.52)	19 (14)	4.32 (2.77–5.22)	0.34
*ARID1A* LoF mutation	10 (8)	2.52 (1.26–2.88)	13 (11)	3.46 (2.60–4.32)	0.83
*CREBBP/EP300* LoF mutation	17 (14)	2.52 (1.26–2.52)	10 (7)	5.53 (3.3–6.05)	0.10
*KMT2D* LoF mutation	7 (6)	1.26 (1.26–2.26)	6 (4)	3.74 (2.31–7.05)	0.78
*SETD2* LoF mutation	4 (3)	2.63 (0.95–4.00)	3 (2)	6.92 (6.23–6.92)	0.71

## Data Availability

The data presented in this study are available on request from the corresponding author. The data are not publicly available due to the requirement to share the data with relevant, approved researchers as stipulated in the ethical approval.
